# Vertebral artery injury due to air rifle: A case report

**DOI:** 10.1016/j.amsu.2021.01.097

**Published:** 2021-02-05

**Authors:** Harmantya Mahadhipta, Muhammad Alvin Shiddieqy Pohan, Andryan Hanafi Bakri

**Affiliations:** a Orthopaedic Surgeon, Tangerang District General Hospital, Tangerang, Indonesia; b General Practitioner, Tangerang District General Hospital, Tangerang, Indonesia

**Keywords:** Case report, Gunshot injury, Cervical trauma, Spinal cord injury, Brown-sequard syndrome, Vertebral artery injury

## Abstract

**Introduction:**

This case report presents a rare case of vertebral artery and spinal cord injury due to air rifle pellet.

**Case presentation:**

A previously healthy 19-year-old male was shot on his left neck incidentally during recreational air rifle game. He was taken to the other hospital before being referred to our hospital.

**Clinical findings and investigations:**

The patient presented with total loss of motoric function on his left side of the body together with sensoric function on the contralateral side from the level of C5 and below. Signs of stroke were also spotted on the patient's face. The cervical plain radiograph and CT scan were carried out preoperatively to depict pellet fragments. Meanwhile, the CT angiography which was commenced postoperatively revealed the left vertebral artery injury.

**Interventions:**

Surgery comprising of pellet fragments removal, decompression and posterior stabilization of the cervical spine was carried out to retrieve the pellet fragments, which were embedded at the posterior epidural space.

**Relevance and impact:**

Our findings were consistent with the vertebral artery injury and Brown-Sequard syndrome. Hence, these clinical entities should be considered in the setting of penetrating cervical trauma.

## 1 Introduction

Cervical gunshot injury is a rare, but potentially harmful condition due to the presence of numerous vital structures located at the neck region. Structures, such as trachea, carotid artery, spinal cord or vertebral artery might be damaged by the trajecting bullet. This highly concerning injury can occur either in military or civilian setting as a result of accidental, homicidal or even suicidal event [1,2]. The type of bullet, high and low velocity, will determine its mechanism of injury which subsequently predicts the damage afflicted. While high velocity bullet is more likely to follow predictable passage, low velocity bullet tends to have a more unpredictable pathway [1,3]. One of the examples of low velocity bullet is the air rifle which use is gaining popularity recently. Labeled as a recreational gun which is commonly used for hunting and other shooting games, it has potential to be a lethal weapon. Some reported cases from the United States, India and European countries have revealed lethal injuries, or even deaths, due to the direct strike towards important anatomical structures, particularly around the head, neck and chest region [[4], [5], [6], [7]]. The incidence of injury caused by air rifle in Indonesia is not precisely known. Nevertheless, the exact number of cases might be considerably significant due to the lack of monitoring of its use among the civilians.

The vertebral artery injury (VAI), although rarely happens, is a catastrophic injury which follows either blunt or penetrating trauma. In most cases, motor vehicle accident and fall account for the majority of the cause of injury to this structure [8]. VAI caused by gunshot is somewhat rare, but has the great impact due to its destructive nature. Several reports revealed the injuries caused by high energy rifles, with stroke or even death ensued due to vertebral artery damage with concomitant spinal cord injury [12]. By far, the reported injury caused by air rifle is extremely scarce. Furthermore, the fact that the air rifle pellet is considered as low velocity makes it way more interesting to be discussed.

The presence of pellet fragment in the spinal canal mandates fastidious retrieval to avoid deadly complications. Albeit, there is still no single approach method that has been proven to be superior to another in order to retrieve foreign body from this precarious canal, with some reported cases implemented either anterior or posterior approach [9,10]. Therefore, in this case illustration, we aim to present a case of 19-year-old male who sustained VAI resulting from air rifle injury and underwent pellet fragment retrieval. This case report has been reported in line with the SCARE criteria [13].

## 2 Case presentation

**Fig. 1:**
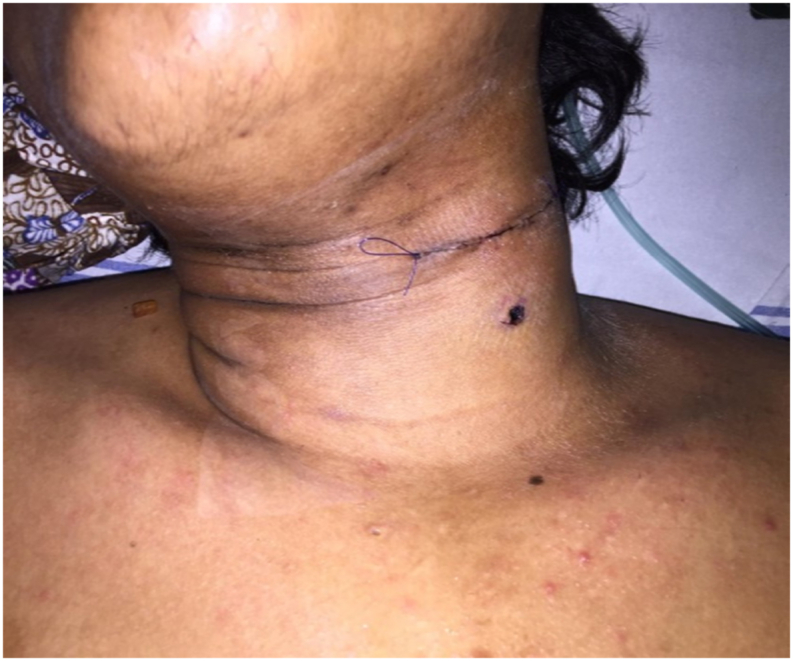
Entry wound of the pellet (round) and sutured post operative wound.

A previously healthy 19-year-old male was shot on his left neck incidentally by his friend during recreational air rifle game from the range of approximately 2 m. He was taken to the nearest local hospital immediately to get his wounded neck treated by a surgeon and was admitted in the hospital ward for 3 days. Subsequently, the patient was referred to our hospital for his left hemiparesis complaint, which he recalled that happened shortly after the incident. The patient's previous drug history, familial history and psychosocial history were unremarkable. On physical examination at our hospital, the patient was fully alert and hemodinamically stable. General examination of the neck discovered sutured post-operative wound sized 5 cm as well as the entry point of the pellet sized 0.5 cm on the anterolateral of the neck at the level of thyroid cartilage (Fig. 1). Neurological examination revealed total loss of motoric function on his left side of the body together with sensoric function on the contralateral side from the level of C5 and below, with an intact bulbocavernosus reflex and perianal sensation. There was also facial lateralization characterized by ptosis and eyebrow as well as lip drooping on the left side.

**Fig. 2:**
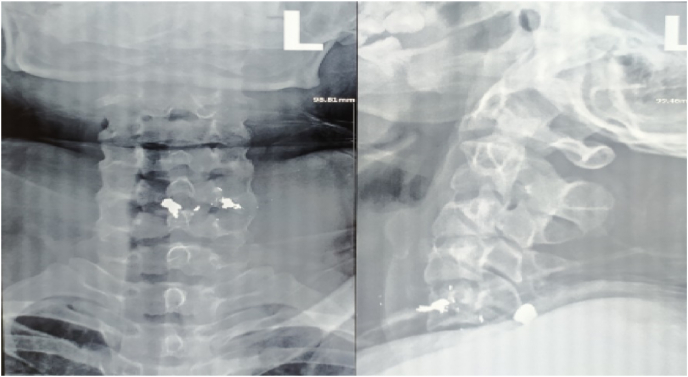
Initial plain cervical radiograph.

**Fig. 3:**
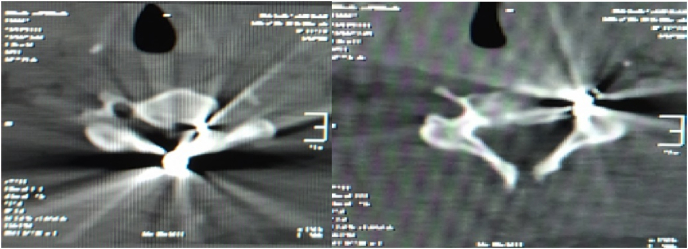
Axial plane of cervical CT scan depicting the position of the pellet fragments.

Initial imaging study of cervical plain radiograph revealed scattered pellet fragments at the level of C5-6 (Fig. 2). Therefore, we conducted CT scan for further ancillary study and revealed that foreign bodies with metallic density found at the left side of C5-6 level and suspicion of right laminar fracture of C5. In addition, the metallic foreign bodies were discovered both within the spinal canal of C5 and in close proximity to the vertebral artery foramen (Fig. 3). Thus, the surgery comprising of pellet fragments removal, decompression and posterior stabilization of the cervical spine was planned immediately.

**Fig. 4:**
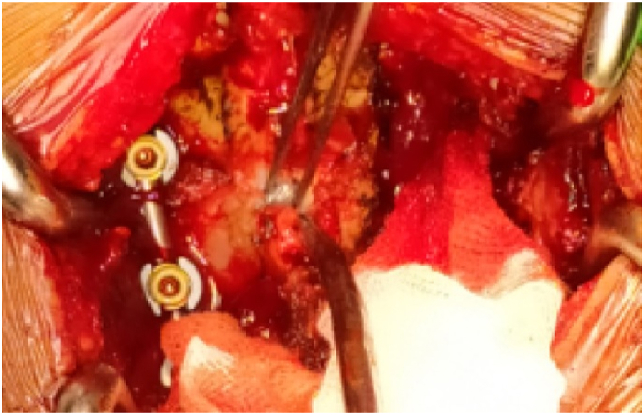
Intra-operative condition depicting the presence of pellet fragment at the duramater (pinched by the forcep).

**Fig. 5:**
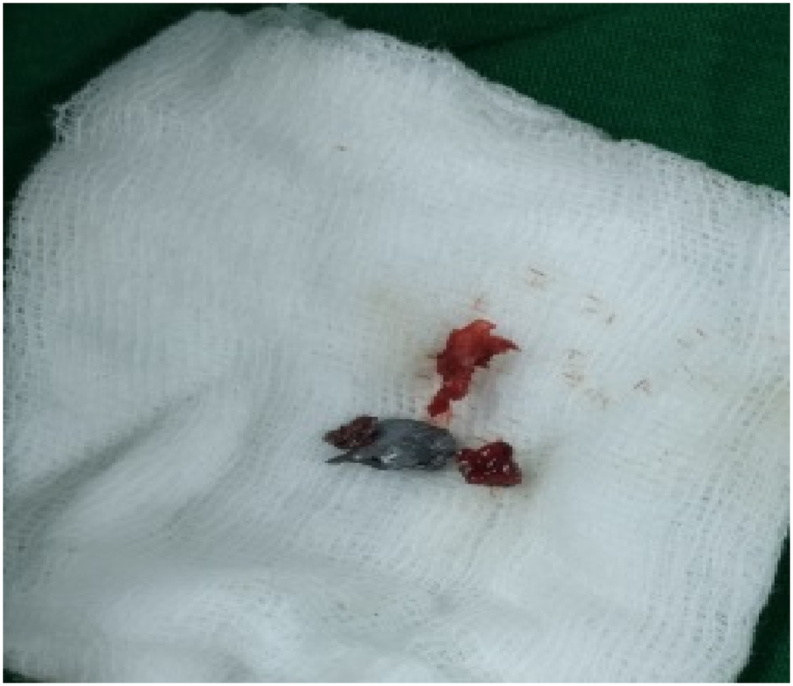
Pellet fragments.

**Fig. 6:**
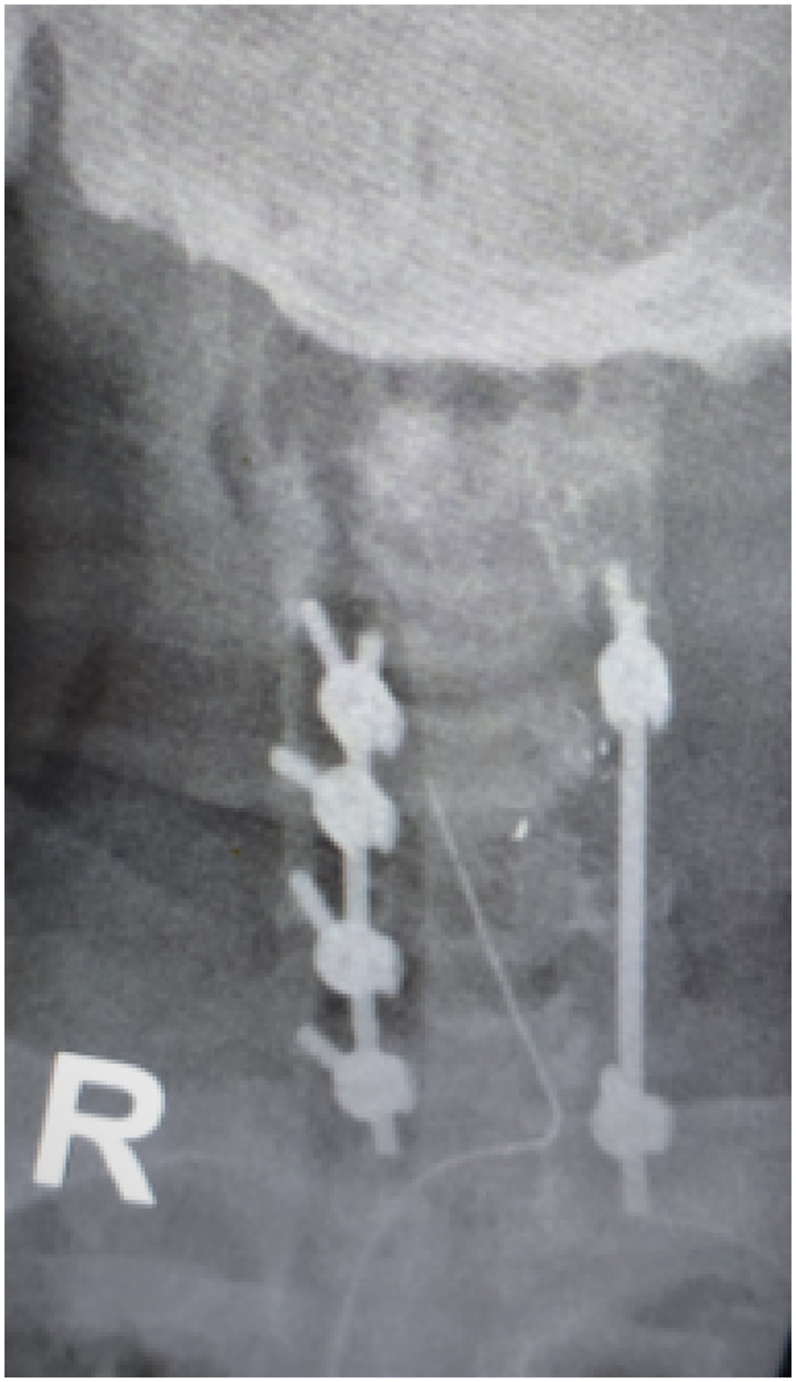
Post-operative plain cervical radiograph.

**Fig. 7:**
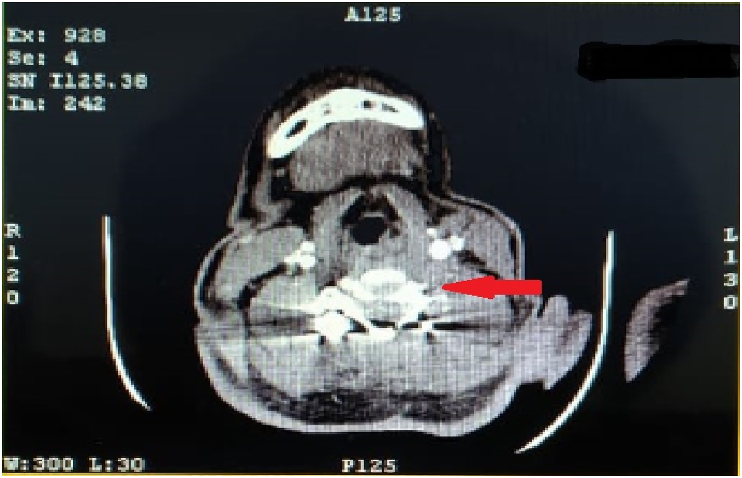
Post-operative CT angiography of the cervical depicting the unpresence of left vertebral artery (red arrow). (For interpretation of the references to colour in this figure legend, the reader is referred to the Web version of this article.)

The surgery, conducted by first author who is an orthopaedic spine surgeon, was brought by using posterior midline approach of the cervical region to expose the posterior column of the vertebrae. Lateral mass screws and rods were installed at the level of C4-7 followed by decompression, only to reveal that a pellet fragment was embedded at the posterior epidural space at the level of C5 with dural laceration (Fig. 4). Afterwards, the fragment sized 0.5 cm was delivered and the exploration by using the image intensifier (C-arm) was proceeded in order to search the other fragments. The other fragments were discovered at the vertebral artery foramen with concomitant vertebral artery transection. Then, the transected artery was packed with bone wax. During this further exploration, we succeeded to deliver 2 other fragments sized 0.5 and 0.4 cm (Fig. 5). Post-operatively, plain cervical radiograph (Fig. 6) and CT angiography were commenced. The latter revealed that the left vertebral artery was completely transected at the level of C4 (Fig. 7). Consequently, incomplete spinal cord injury (SCI) because of the left VAI due to penetrating injury caused by air rifle pellet was marked as the working diagnosis.

## 3 Discussion

Vertebral artery is a branch of subclavian artery which ascends through the cervical region and ends up forming basilar artery [8,11,14]. It resides in the transverse foramen, in which it is well protected. Injury to the vertebral artery occurs unilaterally in most cases and might be a result of either spontaneous or traumatic condition [15,16]. While the spontaneous injury is well recognized and characteristically managed by anticoagulant, endovascular therapy to arterial repair, the traumatic injury is far less well understood in terms of natural course and treatment [16,17]. The cause of traumatic injury can be from blunt or sharp trauma which disrupts the intactness of vessels’ integrity. VAI resulting from the former mechanism can be due to hyperextended, hyperflexed or combined extreme movements to the cervical spine. Whereas, the latter can be produced by sharp objects penetration or gunshot injury [14]. In our case, the injury is inflicted by air rifle, a potentially lethal low velocity rifle that is frequently used in recreational shooting games. Contrary to the firearm gun which uses the energy generated from the burned gunpowder, the air rifle utilizes compressed air or carbon dioxide to launch the projectile with the traveling speed up to 200 m/s. According to Di Maio et al. the threshold of 70 m/s is needed for a projectile to penetrate the human skin. Therefore, with the velocity up to three times greater than the threshold, its pellet has the potential to destroy the skin and critical structures inside the body, including at the cervical region, as noted in our patient. Even though its use has been increasing in recent years, including in Indonesia, the exact incidence of VAI due to air rifle is not well documented. Nevertheless, some reports revealed that children and teenage with male gender are the most frequent victims of accidental shooting by air rifle due to the inept use as well as lack of supervision. This report is consistent with our patient who sustained the injury during a recreational hunting game [5,7].

History taking and physical examination derived from our patient revealed that the pellet was shot from the range of approximately 2 m and left a round entry wound. This finding concurs with the statement by Kuligod et al. who stated that air rifle differed from firearm regarding the entry wound shape. It will yield an oval or round wound, irrespective of the distance between muzzle and the target [18]. Moreover, the range between the muzzle and victim will determine the tissue damage, as classified by Sherman and Parish. In their classification, long (more than 7 yards), medium (3–7 yards) and short (less than 3 yards) range injuries are correlated with the tissue damage, as shorter range is linked to severer injury. Therefore, the injury suffered by our patient is classified as short range injury and massive tissue destruction should be anticipated. In addition, the irregular trajectory created by low velocity bullets will cause the injury pattern become more intriguing and unpredictable [1].

Neurological examination from our patient is consistent with the clinical manifestation of Brown-Sequard syndrome; a form of incomplete SCI. VAI might cause noxious consequence due to either hematoma causing the occlusion or vessel transection. While in some individuals it may show no symptom at all, in other persons its symptoms may be so variable that warrants high index of suspicion. Symptoms, such as headache, vestibular impairment to stroke symptoms can develop following vessel dissection or transection. Anatomically, it may also affect spinal cord, ranging from lateral medulla infarction to spinal cord ischemia [8]. Accordingly, the clinical presentation of the patient in our case might be caused by spinal cord impairment, along with stroke due to VAI as noted by facial lateralization.

Imaging study from plain cervical radiograph revealed the bullet fragments scattered around the level of C5-6. Imaging for the injury at the cervical region should be started with plain radiograph and preceded with other imaging modalities, such as CT scan or magnetic resonance imaging (MRI) as indicated. In this case, due to the presence of metallic object, thus conventional CT scan was ordered to give further information about fragments’ location and confirmed the position of pellet fragments. Moreover, due to awareness of any vascular injury, we conducted the CT angiography examination and revealed the completely transected left vertebral artery. This imaging study was commenced post-operatively due to the emergency surgery which was arranged immediately. The diagnosis of VAI should be contemplated with clinical manifestation of stroke in the setting of trauma. Its existence is then established by imaging study, with digital subtraction angiography (DSA) is the study of choice for diagnosing vascular injury in the head and neck region. Nonetheless, it is invasive, costly and not readily available, including in our hospital. Accordingly, the CT angiography is regarded as the initial screening study for vascular injury of head and neck. Study by Eastman et al. even reveals that this imaging study has both sensitivity and specificity up to 100%, making it is a reliable diagnostic workup for patient with conjecture of VAI [16,19]. While some screening tools, such as Denver, Memphis and Boston criteria are specially designed to raise the index of suspicion for blunt traumatic vertebral artery injury [14], to our knowledge there is still no screening tool for penetrating VAI [11,16,20]. Therefore, the diagnosis of penetrating VAI may be missed in many occasions and it justifies a high index of awareness, as in our case.

VAI is a Treatment for VAI initially is addressed to the life saving, as governed by the Advanced Trauma Life Support (ATLS) protocol. After dealing with the life threatening condition due to the presence of high risk structures, which in this case was provided in other hospital, then the specific intervention is directed to the neurological impairment. There is no clear agreement among experts on the optimal standard management of VAI. Generally, treatment consists of medical management, involving antithrombotic agents, and surgical intervention. While the former is not amenable for penetrating injury, the latter approach is hindered by the complexity of vertebral structures, especially at the cervical region. The open surgery for vertebral artery may be reserved for patients with uncontrollable bleeding or for those who undergo open exploration for spinal condition [8,11]. The complicated management selection is perplexed by the fact that there is still much debate regarding the ideal management of the penetrating VAI. Intention to apply conservative approach due to the challenging surgery is opposed by the attempt to prevent complications caused by the presence of foreign body, such as CSF fistulas, symptomatic adhesions, late spinal cord injury and infection. Meningitis may be caused by the possibility of the pellet fragment to become an infection nidus [6,18,21]. Therefore, in this case we commenced surgical approach, comprising of decompression, posterior stabilization as well as exploration to take the pellet fragments out, in order to prevent further damage to the spinal cord and surrounding structures.

Meticulous debridement and pellet fragment removal is then mandatory to be executed in the case of foreign body presence within spinal canal, which in this scenario was brought through posterior approach. Though there is still no clear evidence regarding the best approach method to expose the spinal canal in the case of foreign body within it, several studies compared the for some other conditions. Generally, approach to the cervical spine comprises of anterior and posterior methods, each of which has its own advantage and disadvantage. Pellets in vertebral artery foramen could be retrieved by either anterior or posterior approach, both by performing foraminotomy, which in our case are the left C5-6. Consideration in choosing posterior approach in this patient is because there is pellet located in the posterior epidural space, which puts retrieval using anterior approach is impossible.

## 4 Conclusion

VAI following air rifle is a rare, but potentially devastating injury. This injury is easily overlooked, thus high index suspicion followed by initial plain cervical radiograph and early CT angiography workup should be applied for patients with penetrating cervical trauma and experience neurological deficit. The treatment for the presence of pellet fragment at the spinal column must include exploration and fragment extraction to prevent further disastrous complications. Posterior approach to remove the pellet fragments can be performed successfully with high vigilance to surrounding structures. Thus, deciding the best approach, anterior or posterior, is determined by the location of foreign body.

## Ethical approval

This is a case report which has been approved by the medical committee to be published on the medical journal.

## Sources of funding

The source of funding of this case report is provided by the authors.

## Author contribution

Harmantya Mahadhipta: Chief surgeon, operator.

Muhammad Alvin Shiddieqy Pohan: Assistant surgeon, case director.

Andryan Hanafi Bakri: General practitioner, editor.

## Guarantor

Harmantya Mahadhipta (main guarantor).

Muhammad Alvin Shiddieqy Pohan.

Andryan Hanafi Bakri.

## Consent

Written informed consent was obtained from the patient for publication of this case report and accompanying images. A copy of the written consent is available for review by Editor-in-Chief of this journal on request.

## Provenance and peer review

Not commissioned, externally peer-reviewed.

## Declaration of competing interest

All authors hereby declare that there is no conflict of interest.
